# Enhancing quantum audio watermarking security through joint verification and certification

**DOI:** 10.1038/s41598-026-36535-w

**Published:** 2026-01-17

**Authors:** Zheng Xing, Chan-Tong Lam, Xiaochen Yuan

**Affiliations:** 1 School of Computer Science, South China Business College, Guangdong University of Foreign Studies, Guangzhou, 510545 Guangdong China; 2 Faculty of Applied Sciences, Macao Polytechnic University, Macao, 999078 S.A.R China

**Keywords:** Engineering, Mathematics and computing

## Abstract

Current quantum audio watermarking schemes prioritize robustness but often overlook critical security vulnerabilities, leaving systems exposed to impersonation and unauthorized use. To address this gap, we propose a dual-security mechanism that synergistically integrates joint verification and certification of watermarks, inspired by the paging seal principle. Our framework incorporates Quantum Error Correction (QEC) coding to enhance resilience against qubit errors and malicious tampering. Experimental results show improved performance: the watermark maintains high imperceptible with SNR> 46 dB under increased embedding rates, and achieves a 62.5% reduction in average BER compared to several state-of-the-art methods at a qubit error probability of 0.10. These results suggest that the proposed approach offers enhanced security and robustness, representing a promising direction for secure quantum audio watermarking.

## Introduction

With the rise of quantum computing in recent years^1,2^, the landscape of data-related research has undergone a significant transformation. The remarkable improvements in computational speed and storage capacity caused by emerging quantum technologies^3,4^ have opened new horizons for various technological research and applications. Quantum computing and quantum information processing exhibit great promise in addressing and improving numerous tasks and applications^5,6^, which has spurred the exploration of leveraging quantum computing techniques to upgrade existing classical based tasks and applications. In the domain of audio-related data protection, traditional audio watermarking techniques^7,8^ have already been the subject of extensive research. However, with the advent of quantum computing, there is a growing need to harness quantum technologies to further strengthen and develop audio copyright protection^9^. While initial efforts in the quantum related cross disciplines have focused on image and video processing^10^, aiming to exploit the vast potential of quantum computing, the application of quantum technologies to audio signals is emerging as a crucial area that warrants in-depth exploration.

As it is crucial to protect quantum data, some techniques for hiding data^11^, such as watermarking and steganography, have also been introduced into the field of quantum computing. In 2015, Wang et al. proposed a quantum representation of digital audio signals (QRDA) in^12^. Then, Yan et al. proposed an FRQA model for the representation of audio signals^13^, and this representation model is based on the Flexible Representation of Quantum Images (FRQI) model^14^. The resulting FRQA model for flexible representation of quantum audio encodes the audio amplitude using two’s complement symbols and integrates the time information into normalized quantum states. Based on FRQA, Chen et al. developed two quantum audio steganography (QAS) protocols^15^, each of which manipulates or modifies the Least Significant Quantum bit (LSQb) of the carrier quantum audio signal. In 2018, Qu et al. proposed an improved quantum watermarking algorithm for quantum audio^16^, utilizing the LSQb for logical consistency and correlation modification to improve the robustness of quantum audio copyright protection watermarking. In 2019, Chen et al. proposed a dual quantum audio watermarking (QAW) scheme^17^, which utilizes quantum Discrete Cosine Transform (qDCT) to achieve secure communication and transmission of quantum audio signals. In the first scheme (QAW-I), quantum multiplication and addition operations are used to facilitate watermark embedding. In the embedding procedure of the second scheme (QAW-II), the main quantum audio signal is partitioned into sub-blocks, each of which is modified using a combination of quantum multiplication and qDCT operations. In 2019, Javad Chaharlang et al. proposed a novel quantum steganography-tampering analysis system^18^ for digital audio signals that can accurately detect audio steganography methods in the context of a quantum communication network. Yoosefi Nejad et al. proposed a quantum audio watermarking method^19^ based on qDCT, embedding the quantum image in the low-frequency component of the main signal. In the same year, Nejad et al.^20^ proposed a new scrambling method for watermarked images in order to optimize robustness and capacity. The scrambled image is then converted into a sequence of quantum bits, and then they embed it into the main quantum audio signal using an embedding key.

In 2020, Mohsen Yoosefi Nejad et al. proposed an enhanced audio watermarking scheme^21^ based on LSB using gray code, in which they used gray codes to prohibit attackers from directly altering the embedded watermark. To increase security, the watermarked image pixel values are modified to scramble. In 2022, Masoumeh Velayatipour et al.^22^ proposed a novel quantum-reversible implementation of echo-hidden audio watermarking based on Quantum Representation of Digital Signals (QRDS)^23^. In the embedding process, some echo frames are generated by modifying the time and amplitude qubits of the main audio frame according to the watermarking qubits. Then, as the sum of the quantum main audio signal and the quantum echo audio signal, the quantum watermarked audio is obtained.

**Fig. 1:**
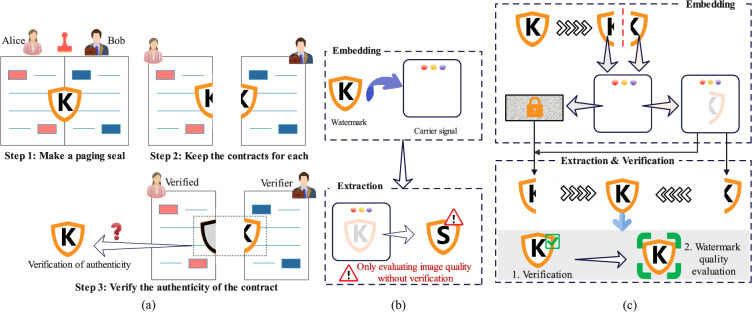
Demonstration of various security mechanisms: **a** the typical process of using paging seals, **b** the general process of current existing quantum watermarking schemes, and **c** our improved secure quantum watermarking model.

In summary, current quantum audio watermarking is mainly based on quantum circuits to realize embedding and extraction of watermark information. Moreover, the evaluation experiments mainly focus on imperceptible and robustness. However, there are problems in two aspects: one is that robustness still needs to be improved, and the other is that the security measures for watermarking need to be considered. Although some methods consider the security of watermark information to prevent leakage, they are unable to prevent and identify infringements. In order to improve the robustness and security of quantum watermarking methods, we utilize the concept of Paging seal for quantum audio watermarking. The paging seal is a widely used security mechanism: it is stamped across two banknotes, and the authenticity of the banknotes is verified by checking whether the two parts can be combined into a complete seal. Figure 1a vividly illustrates the use of paging seals. Figure 1b is the general watermarking algorithm process, which focuses only on the robustness of the watermarking algorithm. Figure 1c is the process of the proposed watermarking algorithm. In our method, instead of considering only the robustness of the algorithm, the security risk of the watermarking algorithm application is also considered.

To prevent copyright impersonation of fake audio or infringing use of watermarks, we utilize the paging seal principle to divide the watermark into an authentication part and a certification part to defend against common infringements. We elaborate the quantum audio watermarking scheme and provide low-complexity embedding and extraction circuits. Furthermore, a series of simulation experiments are used to evaluate imperceptible and robustness. The results show that the watermark is imperceptible at higher embedding rates. Regarding robustness, we utilize the QEC principle to reduce the impact caused by qubit errors, and the experimental results show that our scheme has lower Bit Error Rate (BER) values compared to other methods. Finally, it not only enhances security measures but also has better robustness.

## Results

### Preliminaries

**INEQR**: The Improved Novel Enhanced Quantum Representation of Digital Images (INEQR)^24^ uses the base state of a sequence of qubits to store quantum image information, i.e., a pixel’s grayscale value and corresponding coordinate, into normalized entangled qubits sequence. The representation of INEQR is shown below for a grayscale image of size $2^m\times 2^n$ in the range $\left[ 0,2^{q}-1 \right]$, where $C_{YX}^{i}\in \left\{ 0,1 \right\}$.

1 $$\begin{aligned} | I \rangle = \frac{1}{\sqrt{2^{m+n}}} \sum _{Y=0}^{2^m-1} \sum _{X=0}^{2^n-1} \left( \bigotimes _{i=0}^{q-1} | C_{YX}^{i} \rangle \right) \otimes | Y X \rangle \end{aligned}$$

where ‘$\otimes$’ is the tensor product notation. The pixel grayscale value $\left| C_{YX}^{i} \right\rangle$ is encoded by a sequence of *q* qubits, e.g., eight qubits are used for encoding the pixel values of 8-bit grayscale images. The coordinate information is indicated by two sequences of *n* qubit, denoted as follows.

2 $$\begin{aligned} \left| Y \right\rangle =\left| y_{m-1}y_{n-2}...y_{0} \right\rangle , \left| X \right\rangle =\left| x_{n-1}x_{n-2}...x_{0} \right\rangle \end{aligned}$$

where $\left| y_{i}\right\rangle , \left| x_{i}\right\rangle \in \left\{ \left| 0\right\rangle ,\left| 1\right\rangle \right\}$, and $Y,X\in \left[ 0,2^{n}-1 \right]$. Consequently, for a $2^n\times 2^n$ quantum grayscale image, $2n+q$ qubits are required. $| Y \rangle = |y_{m-1} y_{m-2} \cdots y_0\rangle$ and $| X \rangle = |x_{n-1} x_{n-2} \cdots x_0\rangle$ are the position registers encoding the row and column coordinates. Note that $| Y \rangle \otimes | X \rangle$ is explicitly written to clarify the separation between row and column coordinates, which was previously denoted as $| YX \rangle$ in shorthand notation.

**QRDA**: The Quantum Representation of Digital Audio Signals (QRDA)^12^ shifts the signal amplitude information at any time *t*, integers in the range $\left[ -2^{\frac{q}{2}}, 2^{\frac{q}{2}}-1\right]$ to integers in the range $\left[ 0,2^q-1\right]$ by multiplying by two. At the end of the process, the measured amplitude information is divided into two. The quantum state of the QRDA is prepared as follows for audio $S=\left[ S_0, S_1, \ldots , S_{2^k-1}\right]$ and $S_t \in \left[ 0,2^q-1\right]$.

3 $$\begin{aligned} \begin{aligned}&|S\rangle =\frac{1}{\sqrt{2^k}} \sum _{t=0}^{2^k-1}\left| S_t\right\rangle \otimes |t\rangle \\&\left| S_t\right\rangle =\left| S_t^0 S_t^1 \ldots S_t^{q-1}\right\rangle , \quad S_t^i \in \{0,1\} \\&|t\rangle =\left| t_0 t_1 \ldots t_{k-1}\right\rangle , \quad t_j \in \{0,1\} \end{aligned} \end{aligned}$$

where $\left| S_t\right\rangle$ and $|t\rangle$ are the binary sequences of the amplitude value and the time, respectively.

**QEC**: Quantum error correction (QEC)^25^ and fault-tolerant quantum computing^26^ represent two of the most important theoretical aspects of quantum information processing. It is well-known that the fragility of coherent quantum systems is still a catastrophic obstacle to the development of large-scale quantum computers. The realization of the quantum error correction in 2004 has shown that active techniques^27^ can be employed to alleviate this critical issue. The 3-quantum bit-flip code is the traditional basic quantum error correction code. Essentially, a 3-qubit QEC code encodes a logical qubit into three physical qubits with the property that it can correct a quantum bit-flip error. The two logical ground states $\left| 0\right\rangle _L$ and $\left| 1\right\rangle _L$ are defined as $\left| 000\right\rangle$ and $\left| 111\right\rangle$. The principle and quantum circuits for its encoding and correction of the single quantum bit-flip error can be found in^28^.

### The proposed method

The proposed quantum audio watermarking method aims to embed INEQR-based quantum binary images imperceptibly in quantum audio. Our method mainly consists of a quantum watermark embedding process and an extraction process. The highlight is that we use the paging seal concept to split the watermark into two parts with the watermark paging factor $\alpha$ ($\alpha \ge 1$), one to verify the watermark and one to confirm the copyright. Note that the quantum circuits and simulation experiments in this paper default to the factor $\alpha =1$. In practice, the value of $\alpha$ can be flexibly adjusted as needed.

#### Quantum watermark embedding

**Fig. 2:**
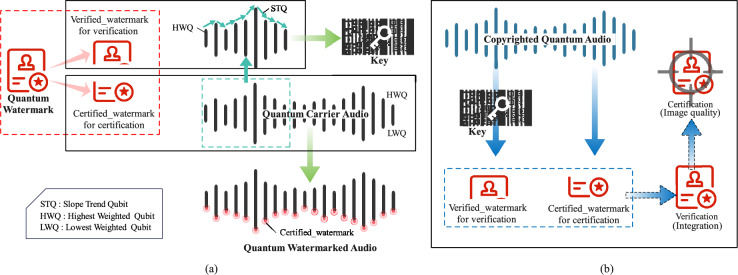
Procedure of the proposed quantum audio watermarking algorithm: **a** watermark embedding, and **b** watermark verification and certification.

Figure 2 presents the whole process of our quantum audio watermarking scheme. Figure 2a illustrates our scheme by embedding the watermark in the Lowest Weighed Qubit (LWQ) of the carrier audio and generating the watermark key to verify the watermark using the Highest Weighed Qubit (HWQ) and Slope Trend Qubit (STQ). The HWQ and LWQ are explained below. For an 8-qubit representation of an audio amplitude in QRDA, the amplitude value is calculated as follows:

4 $$\begin{aligned} \textrm{Amplitude} =128\times \underset{\overline{\mathrm {{{\small HWQ} } } } }{p_7} +64\times p_6+...+ 1\times \underset{\overline{\mathrm {{{\small LWQ} } } } }{p_0} \end{aligned}$$

The qubits with the highest weight is HWQ, and vice versa for LWQ. We propose the concept of STQ by utilizing the difference between the amplitude values of two adjacent audio samples as the slope, so that a quantum audio of length $2^l+1$ can be computed to produce a slope sequence of size $2^l$. These slopes may be positive, negative, or zero, and then we collect their sign to produce slope trend qubits. This is done by setting the corresponding STQ to $\left| 0\right\rangle$ if the sign is ‘+’ or ‘-’, otherwise to $\left| 1\right\rangle$. The STQ is defined as follows:

5 $$\begin{aligned} STQ=\left\{ \begin{matrix} \left| 0 \right\rangle , \left| HWQ(i+1)-HWQ(i) \right| \ne 0 \\ \left| 1 \right\rangle , \left| HWQ(i+1)-HWQ(i) \right| = 0 \end{matrix}\right. \end{aligned}$$

Figure 2b depicts the watermark extraction and dual security mechanism. The key is used to verify the carrier audio and the watermark information extracted from the audio is used to certify the watermark, thus ensuring the legitimacy of the copyright.

**Fig. 3:**
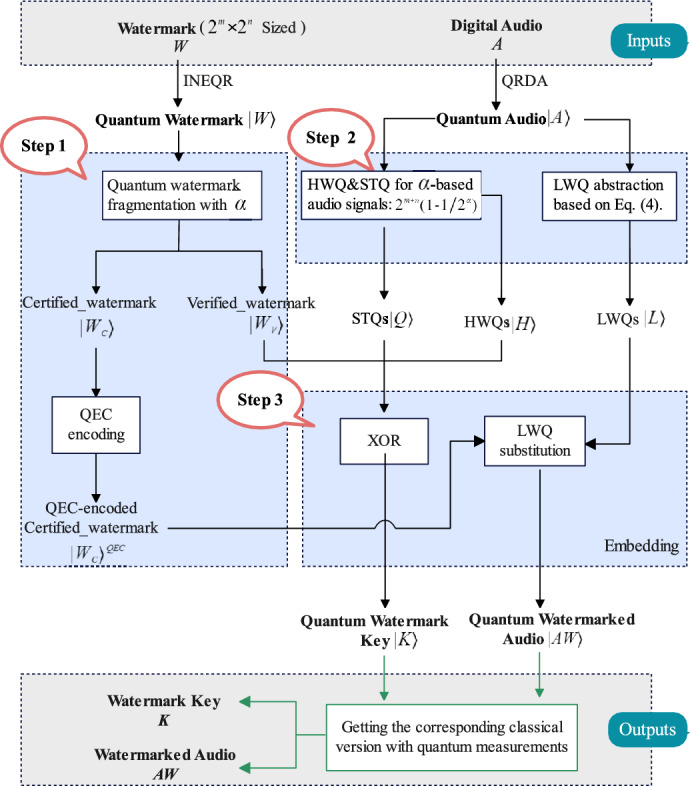
Flowchart of the proposed quantum audio watermark embedding, where $\alpha$ is the watermark paging factor.

Figure 3 shows the embedding flowchart, the inputs are classical watermarked image and digital audio, they go through a quantum state preparation process and a three-step watermarking embedding, and finally the outputs i.e., the watermarked audio and the key are obtained.

**Preparation**: Representation of watermarked images and digital audio as quantum states is essential before watermark embedding. The watermark image *W* is represented as a quantum image $\left| W\right\rangle$ according to the INEQR representation model. Moreover, digital audio *A* is represented as quantum audio $\left| A\right\rangle$ based on QRDA representation.

**Step 1**: The quantum watermark image is fragmented into two parts: one is the verified_ watermark $\left| W_v\right\rangle$, and the other is the certified_watermark $\left| W_c\right\rangle$. The ratio of the two parts of the watermark image is determined by the fragmentation parameter $\alpha \in Z^{+}$. The size of the certified_watermark $S_{\left| W_c\right\rangle }$ is defined as follows:

6 $$\begin{aligned} S_{\left| W_c\right\rangle }=2^m\times 2^n\times \frac{1}{2^{\alpha }} \end{aligned}$$

Then, the size of the verified_ watermark $S_{\left| W_v\right\rangle }$ is calculated as follows:

7 $$\begin{aligned} S_{\left| W_v\right\rangle }=2^m\times 2^n\times (1-\frac{1}{2^{\alpha }}) \end{aligned}$$

Next is the QEC processing of $\left| W_c\right\rangle$ to improve robustness. According to the basic principles of QEC and quantum circuits, we encode the logical information of $\left| W_c\right\rangle$ into a triple-size physical quantum representation, denoted $\left| W_c\right\rangle ^{QEC}$.

**Step 2**: The main purpose of this step is to prepare the key generation $\left| K\right\rangle$ using $\left| W_v\right\rangle$ and the carrier audio, i.e., the calculation of the required STQ sequences $\left| Q\right\rangle$ and HWQs $\left| H\right\rangle$. Note that the length of $\left| Q\right\rangle$ and $\left| H\right\rangle$ is the same as the number of pixels in the verified_ watermark $\left| W_v\right\rangle$, i.e., $2^{m+n} \times (1-\frac{1}{2^{\alpha }})$. When $\alpha$ = 1, the length is $2^{m+n-1}$. They are computed with reference to Eqs. (4) and (5). The LWQ abstraction is then applied to all samples of the entire audio, calculated with reference to Eq. (4).

**Step 3**: Next, we have the embedding process. For $\left| W_v\right\rangle$, we use it to perform the XOR operation with HWQs $\left| H\right\rangle$, STQs $\left| Q\right\rangle$ to make $\left| W_v\right\rangle$ strongly associated with the carrier watermark, thus generating the watermark key $\left| K\right\rangle$. It is worth emphasizing that $\left| K\right\rangle$ not only protects the watermark information but can also be used to verify the authenticity of the watermark. For $\left| W_c\right\rangle ^{QEC}$, we exchange it with LWQ to embed it in the audio, also to minimize the interference of the embedding operation on the original audio. And the length of the LWQ sequence employed is equal to 3/4 size of the quantum audio $\left| AW\right\rangle$. Finally, the quantum watermarked audio $\left| AW\right\rangle$ is obtained. Classical information can be recovered from quantum state information with the help of a finite number of quantum measurements.

**Fig. 4:**
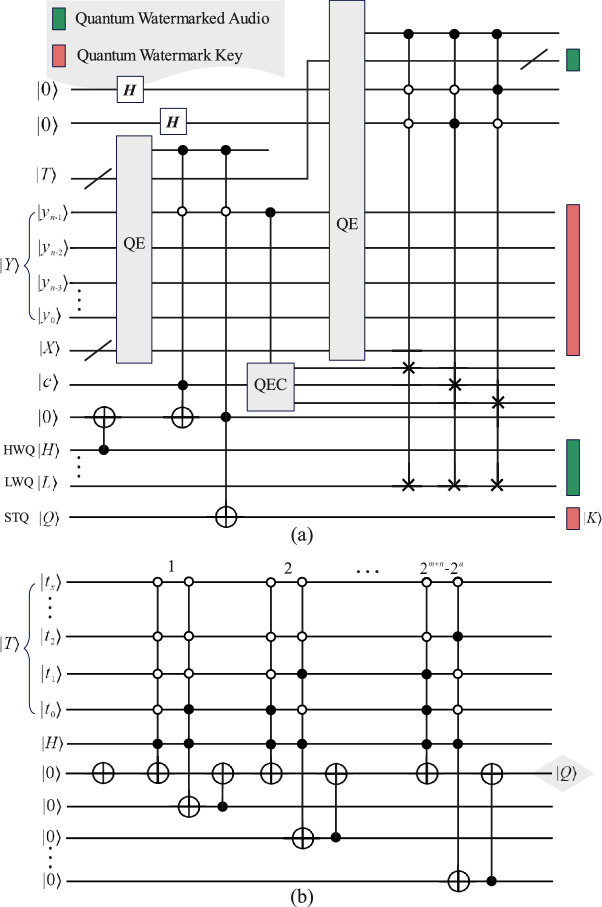
Quantum circuits for watermark embedding. **a** The quantum circuit for embedding, **b** the quantum circuit for calculating SQTs. Note that this is the quantum circuit for an equally segmented watermark image; in case of other segmentation ratios, just modify the number of control qubits in $\left| Y\right\rangle$.

Figure 4 provides the necessary quantum circuits for quantum watermark embedding in order to be implemented on a quantum computer. Figure 4a is the embedding circuit, while Fig. 4b is the STQ computing module circuit. The Quantum Equal (QE)^29^ module is used to compare whether two quantum sequences are equal or not. Only one indicator quantum state is required to know the result, if the result is $\left| 1\right\rangle$ then it means equal, otherwise, it is not equal. The QE in the circuits is applied to maintain the correct order of the qubits.

To better demonstrate the practicality of our solution, security concerns regarding the key are explained as follows. The secret key $|K\rangle$ is generated through a bitwise XOR operation between the verified watermark $|W_v\rangle$ and the concatenated sequences of the carrier audio’s Highest Weighted Qubit (HWQ) $|H\rangle$ and Slope Trend Qubit (STQ) $|Q\rangle$, as defined in Eq. (8):

8 $$\begin{aligned} |K\rangle = |W_v\rangle \oplus (|H\rangle \parallel |Q\rangle ) \end{aligned}$$

where $\oplus$ denotes the XOR operation and $\parallel$ represents concatenation. Consequently, the key length $L_K$ directly corresponds to the number of pixels in the verified watermark $|W_v\rangle$, which is determined by the watermark paging factor $\alpha$ and the original watermark dimensions $2^m \times 2^n$:

9 $$\begin{aligned} L_K = 2^{m+n} \times \left( 1 - \frac{1}{2^{\alpha }}\right) \text { qubits} \end{aligned}$$

For the specific implementation evaluated in this study ($m=n=8$, $\alpha =1$), this yields:

$$L_K = 2^{8+8} \times \left( 1 - \frac{1}{2^1}\right) = 65,\!536 \times 0.5 = 32,\!768 \text { qubits}$$

This substantial key length ensures an exceptionally large key space of approximately $2^{32,\!768}$, making exhaustive search (brute-force) attacks computationally infeasible even with future quantum computing capabilities.

#### Quantum watermark extraction

**Fig. 5:**
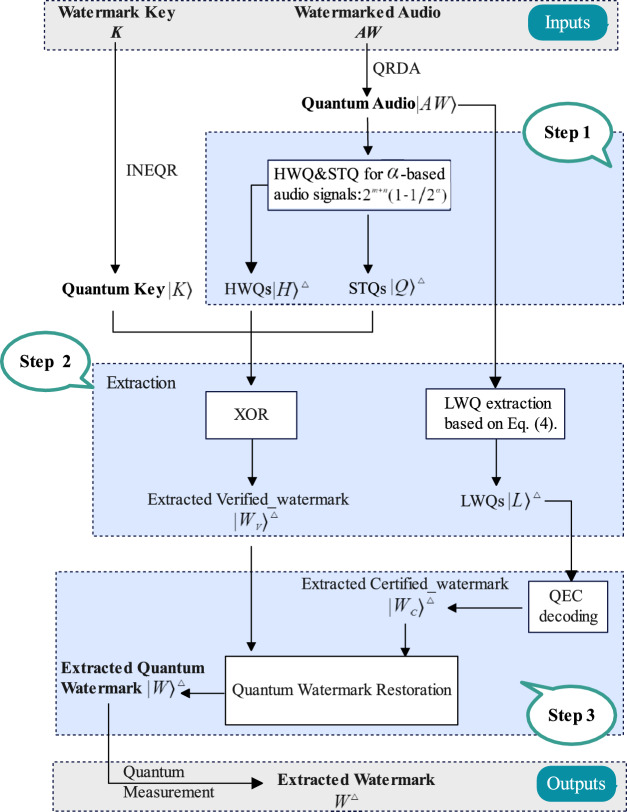
Flowchart of the proposed quantum audio watermark extraction, where $\alpha$ is the watermark paging factor.

Essentially, the extraction process of quantum audio watermarking is the inverse process of embedding. In general, the quantum watermark extraction process requires the watermark key *K* and the watermarked audio *AW*. Extract the verified_ watermark and the certified_watermark, respectively, and then merge them to get the extracted watermark. Figure 5 illustrates the extraction flowchart of the proposed quantum audio watermarking method.

**Step 1**: First, in order to extract the verified_ watermark $\left| W_v\right\rangle ^{\bigtriangleup }$ utilizing the key $\left| K\right\rangle$, we need to compute the needed STQ sequence $\left| Q\right\rangle ^{\bigtriangleup }$ of the watermarked audio $\left| AW\right\rangle$. Note that the length of $\left| Q\right\rangle ^{\bigtriangleup }$ is

10 $$\begin{aligned} 2^{m+n}\times (1-\frac{1}{2^{\alpha }}) \end{aligned}$$

Since we default to $\alpha = 1$, then the length of $\left| Q\right\rangle ^{\bigtriangleup }$ is $2^{m+n-1}$.

**Step 2**: Subsequently, we need to extract the verified_ watermark $\left| W_v\right\rangle ^{\bigtriangleup }$, and obtain the LWQs sequence $\left| L\right\rangle ^{\bigtriangleup }$ based on Eq.(4). We use the LWQ sequence of $\left| AW\right\rangle$ to obtain the extracted $\left| W_c\right\rangle ^{\bigtriangleup }$ by applying the QEC decoding circuit. Then, we perform the XOR operation of $\left| K\right\rangle$ with the sequences of $\left| H\right\rangle ^{\bigtriangleup }$ and $\left| Q\right\rangle ^{\bigtriangleup }$ by position, respectively, and then we obtain the result as $\left| W_v\right\rangle ^{\bigtriangleup }$.

**Step 3**: Finally, we use the LWQ sequence of $\left| AW\right\rangle$ to obtain the extracted $\left| W_c\right\rangle ^{\bigtriangleup }$ by applying the QEC decoding circuit. An INEQR empty binary image $\left| E\right\rangle$ of size $2^m\times 2^n$ with all pixel values $\left| 0\right\rangle$ is prepared, which is used to store the extracted $\left| W_v\right\rangle ^{\bigtriangleup }$’s and $\left| W_c\right\rangle ^{\bigtriangleup }$’s information to obtain the watermark image $\left| W\right\rangle ^{\bigtriangleup }$.

**Fig. 6:**
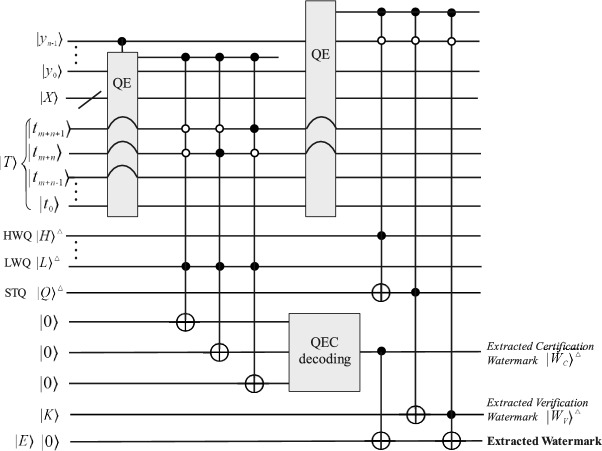
Quantum circuit for watermark extraction.

Figure 6 is the quantum circuit for quantum watermark extraction for quantum computing. The extracted quantum watermark image can be retrieved as a digital image by a finite number of quantum measurements. The required measurements used to retrieve the image are given in^12^.

#### Complexity and capacity analysis

The complexity of a quantum circuit depends on the number of elementary quantum gates used. For all complex unitary operations on an arbitrary number of qubits, all n-controlled quantum gates can be represented as a combination of one- and two-bit quantum gates^30^. Thus, the circuit complexity of any one-bit quantum gate or two-bit quantum gate is 1. The complexity of a quantum circuit is determined by the number of these quantum logic gates. Moreover, this is a common approach when designing quantum circuits that introduce auxiliary qubits $\left| 0\right\rangle$ and $\left| 1\right\rangle$. Literature^31^ states that an n-controlled-NOT (n-CNOT) gate (n$\ge$3) is equivalent to 2(n – 1) Toffoli gates and 1 controlled NOT gate with enough auxiliary qubits, whereas a Toffoli gate can be simulated by six controlled-NOT gates.

The complexity of the quantum circuit used for embedding in Fig. 4 is analyzed as follows. Figure 4a contains 2 QEs, the first QE compares two m+n qubit sequences, and the second QE compares two m+n+2 qubit sequences. According to^29,30^, the quantum circuit complexity of the first QE is $14\times (m+n)-11$; in addition, the complexity of the second QE is $14\times (m+n+2)-11$. Besides, the QEC requires two CNOTs with a complexity of 2. The other quantum gates are 2 and H gates, a CNOT, two 3-CNOT, and three 3-CSWAP. Note that the complexity of an n-CNOT is $14\times n-11$ and that a SWAP quantum gate can be decomposed into 3 CNOTs. Therefore, a 3-CSWAP is equivalent to a 4-CNOT. In summary, the complexity of the embedded quantum circuit is as follows.

11 $$\begin{aligned} \begin{aligned}&14\times (m+n)-11+14\times (m+n+2)-11+1+2+2\\&+2\times 14\times 3-11+3\times 14\times 4-11\\&=28(m+n)+236 \end{aligned} \end{aligned}$$

Similarly, the quantum circuit in Fig. 4b has a total of $2^{m+n-1}$ STQ computations, and one computation of the circuit consists of one CNOT gate and two 5-CNOT gates, the complexity is 119. Thus, the complexity of the STQ quantum circuit is calculated as $(2^{m+n-1})\times 119+1$.

For watermark extraction, the extraction circuit in Fig. 6 contains two QE, three 4-CNOTs, three 3-CNOTs, one CNOT, and the QEC decoding circuit. The QEC requires four CNOTs, hence, the complexity is calculated as follows. The first QE compares two $m+n-1$ qubit sequences, while the second compares two $m+n$ qubit sequences. According to the rules of decomposition of quantum gates, the complexity of the quantum watermark extraction circuit is as follows.

12 $$\begin{aligned} \begin{aligned}&14\times (m+n-1)-11+14\times (m+n)-11+4\\&+1+3\times (14\times 4-11)+3\times (14\times 3-11)\\&= 28(m+n)+197 \end{aligned} \end{aligned}$$

In summary, the embedding complexity of the quantum circuit of the proposed quantum audio watermarking scheme is $O(m+n)\in O(l)$ without taking into account the pre-preparation of the STQ, while the extraction complexity is $O(m+n)\in O(l)$.

The embedding capacity in quantum steganography is defined as the ratio of the number of secret qubits $N_s$ to the number of carrier signal samples $N_c$. The unit is bits per sample. The expression is as follows.

13 $$\begin{aligned} Capacity=\frac{N_s}{N_c } \end{aligned}$$

In the proposed scheme, the size of the watermark image is $2^m\times 2^n$, and we embed $\left| W_v\right\rangle$. Note that the size of the carrier audio is lager than $4\times 2^{m+n} \times (1-\frac{1}{2^{\alpha }})$. Since the embedding capacity is expanded by the application of QEC, the embedding capacity is calculated as follows.

14 $$\begin{aligned} Capacity\le \frac{3\times 2^{m+n} \times (1-\frac{1}{2^{\alpha }})}{4\times 2^{m+n} \times (1-\frac{1}{2^{\alpha }})} = 0.75 \end{aligned}$$

Note that due to the limitations of the QRDA audio model, i.e., the presence of redundancy, this results in an embedding capacity of 0.75 for our method in quantum audio, but if redundancy is not taken into account, our embedding capacity is maximized to 1.

### Experiments and results

**Fig. 7:**
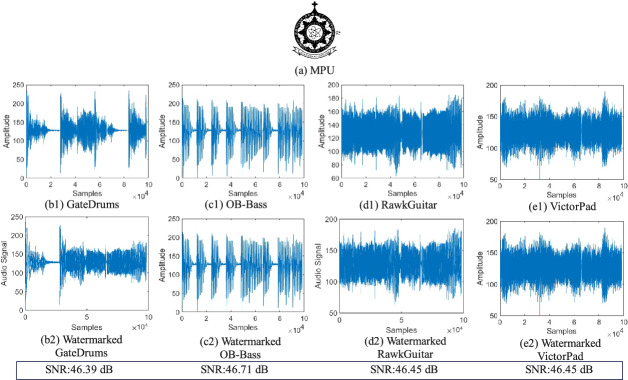
Examples of the four audio carriers and the corresponding watermarked audios, with watermark ’MPU’ of size $256\times 256$ embedded. **a** Watermark ’MPU’, (**b1**)–(**e1**) Four carrier audio segments, and (**b2**)–(**e2**) show the watermarked audios, the corresponding SNR values are calculated as 46.39dB, 46.71dB, 46.45dB, and 46.45dB, respectively.

The proposed methods were simulated using MATLAB 2020b on a conventional computer with an Intel(R) Core(TM) i7 CPU 2.30GHz 16GB RAM, as quantum computers are still in early stages and not yet universally available. Figure 7 shows the material used for the simulation experiments, including four pieces of open-access digital audio permanently archived in Zenodo (DOI: 10.5281/zenodo.18072766). A binary image of the Macau Polytechnic University (MPU) logo of size $256\times 256$ is used as the watermark. The audio size used for the simulation experiments is $3\times 2^{15}$, but due to the limitations of the QRDA quantum audio model, i.e. the presence of invalid redundancy, the audio length in the quantum system is $2^{17}$. Figure 7 shows the portion that is used while ignoring invalid redundancy. All the results in this paper follow the quantum system.

In this section, we assess the imperceptible of the watermark by evaluating the quality of the watermarked audio. Subsequently, we perform the security and robustness experiments. To visualize and quantify the results, we utilize a variety of metrics, including Signal to Noise Ratio (SNR)^32^, Bit Error Rate (BER)^33^, Accuracy^34^ and Intersection over Union (IoU)^35^ to evaluate the quality of the extracted watermark.

#### Imperceptible evaluation

Good imperceptible ensures that the quality of the signal is not significantly affected so that it is imperceptible to the listener. We used the SNR to assess the imperceptible of the proposed method. This metric is defined as the ratio of signal power to background noise power in dB.

Figure 7 collects the results of quantum watermarked audio signals in terms of SNR using the proposed method. From the results, it can be seen that the SNR of the four audio segments after embedding the watermark ‘MPU’ is close to each other, and all of them remain above 46 dB. This means that the embedded watermark does not have a significant effect on quantum audio, which is undetectable to the human ear.

#### Security analysis

The proposed quantum audio watermarking scheme in this paper not only realizes the embedding and extraction of watermarks, but also considers the security of the scheme, i.e., it has the ability to check the extracted watermarks and reduces the risk of copyright infringement.

**Fig. 8:**
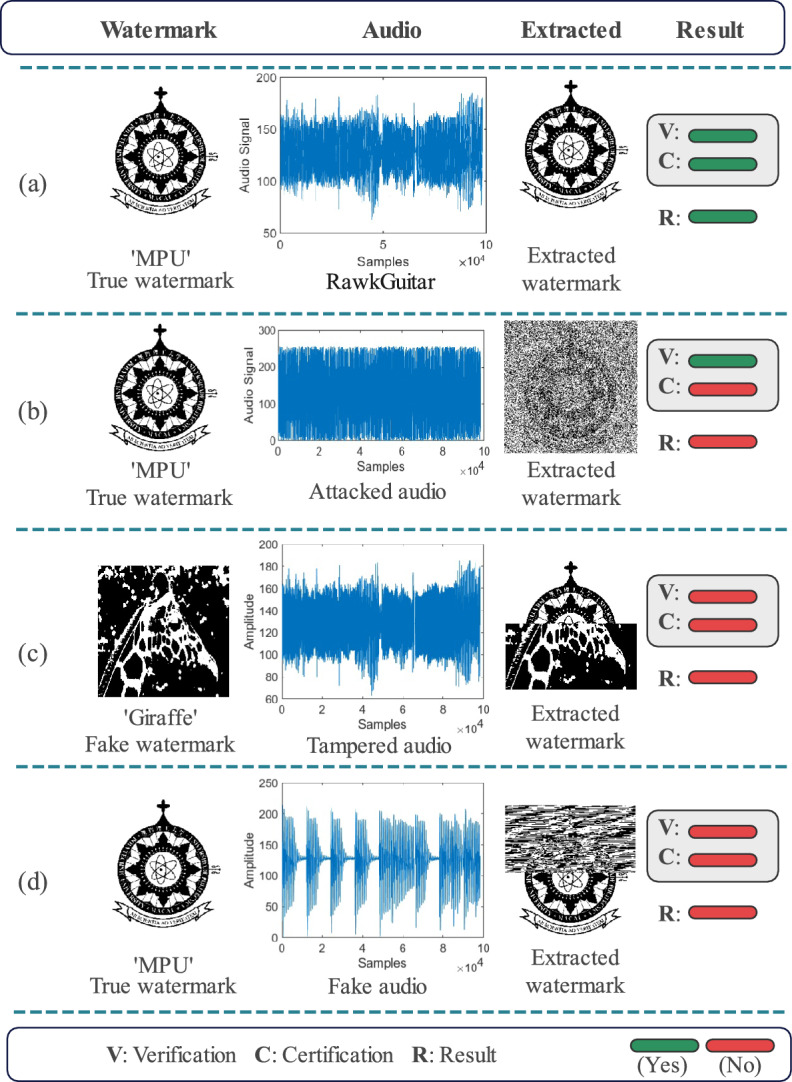
Demonstration of security assessment in various aspects: **a** authentic copyright without security risks, **b** a significant attack that invalidates the copyright, **c** attempted copyright tampering, and **d** attempted copyright impersonation.

Figure 2 demonstrates the proposed quantum audio watermarking scheme with a dual security mechanism in the watermark extraction process. The correctness of the audio and watermark is first verified by the extracted two-part watermark, and then the quality of the watermark image is evaluated to certify copyright validity. In terms of security, benefitting from our watermarking mechanism, the proposed scheme effectively prevents three key risks: watermark forgery (unauthorized creation of fake watermarks to impersonate copyright ownership), watermark tampering (unauthorized modification of copyright information), and watermark misappropriation (unauthorized application of others’ watermarks to one’s own work). Additionally, since the watermark is split and partially encrypted, any attacker can access at most a portion of the watermark information. This ensures the watermark image cannot be fully reconstructed or abused; even if partially obtained, it cannot perfectly impersonate the original copyright as explained below. To demonstrate the security of our scheme, we performed the following evaluation experiments separately: tampering watermarks, forged and stolen watermarks, and imitation watermarks that are edited to make them weakly related to the copyrighted work. In the experiments, we use audio ‘RawkGuitar’ as the carrier and ‘MPU’ as the watermark. Then we use ‘OB-Bass’ as the fake audio and the USC-SIPI^36^ image ‘Giraffe’ as the fake watermark. Figure 8 shows the results of the above experiments.

From the results, it is clear that only Fig. 8a is authenticated successfully, while all other infringements are authenticated failures based on the dual security mechanism. Therefore, our scheme prevents illegal acts of copyright infringement by tampering with watermarks, counterfeiting copyrights, and misappropriating watermarks. It is worth mentioning that Fig. 8c and d confirm the security of tampering with watermarks and misuse of watermarks to impersonate copyrights. Since our embeddings are LWQ substitutions, we consider it an extreme case that the watermark information is completely stolen or replaced to impersonate copyright. However, the generated key is strongly correlated with the carrier audio and watermark information and is inaccessible to the impostor. The final extracted watermark cannot be verified and confirmed, and thus the infringement fails.

Beyond the dual verification-certification mechanism, our scheme’s security fundamentally relies on the cryptographic strength of the secret key $|K\rangle$. With a key length of 32,768 qubits for our evaluated configuration ($\alpha =1$), the key space comprises approximately $2^{32,\!768}$ possible combinations. Even assuming a hypothetical quantum computer capable of testing $10^{30}$ keys per second (far beyond current capabilities), an exhaustive search would require approximately $10^{9856}$ years–rendering brute-force attacks completely impractical. Regarding the statistical analysis resistance, the key $|K\rangle$ exhibits strong pseudorandom characteristics due to its generation process: (1) Dual Dependency: $|K\rangle$ depends on both the secret watermark $|W_v\rangle$ and carrier audio features ($|H\rangle$ and $|Q\rangle$), preventing statistical inference from either component alone. (2) Non-linearity: The XOR operation combined with the STQ computation (Eq. (5)) introduces non-linear relationships that resist linear cryptanalysis. (3) Key Uniqueness: Each carrier audio-watermark pair produces a distinct key due to the audio-dependent HWQ and STQ sequences, preventing key reuse across different content. In terms of Known-plaintext attack resistance, even if an adversary obtains both the watermarked audio $|AW\rangle$ and the extracted watermark $|W\rangle ^\triangle$, they cannot directly compute $|K\rangle$ because:

$$|K\rangle = f(|W_v\rangle , |H\rangle , |Q\rangle ) \ne g(|AW\rangle , |W\rangle ^\triangle )$$

where *f* and *g* denote the key generation and extraction functions respectively. The one-way nature of the STQ computation and the irreversible embedding process prevent backward derivation of $|K\rangle$. Moreover, in practical deployment, the key can be further protected using established cryptographic techniques^37–39^. These cryptographic properties^40^ ensure that our watermarking scheme maintains robust security even against adversaries with partial knowledge of the system.

#### Robustness analysis

In terms of robustness, we consider the performance under quantum channels. Since audio signals in quantum channels are mainly affected by qubit flip, our simulation experiments are the results of a qubit flip channel with different error probabilities (1-p). To evaluate the quality of extracted watermarks under different probabilities of qubit flip, we use the metrics BER, IoU, and Accuracy to quantify the results. The closer BER is to 0, it indicates high quality of the watermark. And the closer the IoU and Accuracy are to 1, the better the watermark quality.

Table 1 collects the extracted watermark quality in terms of 1, 2, and 3 for four pieces of watermarked audio at different qubit flip probabilities: 1-p $\in \{0.01,0.02,0.05,0.10\}$. When 1-p=0.1, the average BER, IoU, and Accuracy values are 0.063, 0.915 and 0.935 respectively. This means that the robustness of the qubit flip channel performs well.

**Table 1:** Results of watermark extraction in qubit flip channel in terms of BER, IoU and accuracy.

1-p	BER Values				IoU Values				Accuracy Values			
	Drums	OB-Bass	Guitar	VicPad	Drums	OB-Bass	Guitar	VicPad	Drums	OB-Bass	Guitar	VicPad
0.01	0.005	0.005	0.004	0.005	0.993	0.993	0.994	0.994	0.995	0.995	0.995	0.995
0.02	0.010	0.010	0.010	0.011	0.986	0.986	0.986	0.987	0.989	0.989	0.990	0.990
0.05	0.027	0.027	0.028	0.027	0.963	0.962	0.963	0.961	0.972	0.971	0.972	0.970
0.10	0.062	0.063	0.063	0.064	0.914	0.915	0.915	0.916	0.934	0.935	0.935	0.935

**Fig. 9:**
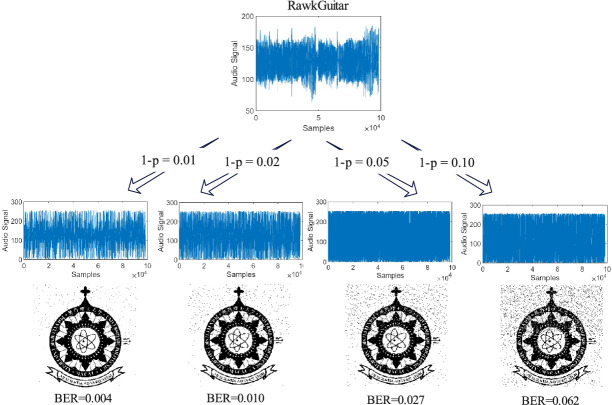
Demonstration of watermark extraction from watermarked ’RawkGuitar’ in qubit flip channel, with 1-p being 0.01, 0.02, 0.05, and 0.1, respectively. The extracted watermark is respectively shown, and the corresponding BER values are calculated as 0.004, 0.010, 0.027, and 0.062 respectively.

From the results, when 1-p = 0.01, the average BER, IoU and Accuracy values are below 0.01, above 0.993, and above 0.994, respectively. This demonstrates good robustness under the qubit flip channel. Figure 9 shows the extracted watermarks with different 1-p being 0.01, 0.02, 0.05, and 0.1 along with the corresponding BER values.

To clarify the performance characteristics of these representative studies, we now elaborate on each work’s specific results in terms of embedding capacity and audio imperceptibility. In 2019, the LSB embedding method proposed by Nejad et al. (^20^) and the dual-watermark method proposed by Chen et al. (^17^) both achieved an embedding capacity of 0.50 bits/sample, but their SNRs were 46.15 dB and 36.00 dB, respectively–with the latter showing significantly lower audio imperceptible than the former. In 2020, Nejad et al. further proposed a blind extraction method (^19^) and an enhanced scheme (^21^): the blind extraction method had a reduced capacity of 0.25 bits/sample and an SNR of 43.51 dB, while the enhanced scheme restored the capacity to 0.50 bits/sample and improved the SNR to 58.87 dB, achieving a notable optimization in imperceptible at that time. In 2022, the quantum echo-hiding method proposed by Velayatipour et al. (^22^) achieved the current highest SNR of **60.31 dB**, but at the cost of reducing the capacity to 0.0625 bits/sample.

In contrast, the proposed method exhibits the optimal performance in terms of embedding capacity, reaching 0.75 bits/sample–12 times higher than that of Velayatipour’s method (2022) and 1.5 times higher than that of Nejad’s enhanced scheme (2020). Regarding audio imperceptible, the average SNR of the proposed method is *46.50 dB*, which, although lower than the 60.31 dB of Velayatipour’s method, is higher than that of Nejad’s method (2019, 46.15 dB) and Nejad’s blind extraction method (2020, 43.51 dB). This confirms that the proposed method still maintains a high level of imperceptible while achieving a better balance between high capacity and high imperceptible.

To test the performance of our watermarking scheme under higher quantum bit error rates, we conducted an extensive ablation study by varying the watermark paging factor $\alpha$ and analyzing the corresponding Bit Error Rate (BER) at qubit flip probabilities ranging from 0.10 to 0.20. As illustrated in Fig. 13, our method demonstrates consistent and gradual BER growth across all tested $\alpha$ values without abrupt degradation. The results reveal several important findings: First, for all $\alpha$ settings (ranging from 1 to 5, corresponding to capacities from 0.75 to 0.046875), the BER increases smoothly with rising qubit flip probabilities. Notably, the growth ratio from 0.10 to 0.20 remains relatively consistent across different $\alpha$ values, ranging from approximately 3.5x to 3.7x. This indicates that our quantum error correction mechanism maintains its protective efficacy even at elevated error rates. Particularly, at $\alpha =1$ (Capacity=0.75), the BER rises from 0.0140 at p=0.10 to 0.0517 at p=0.20. For the most conservative setting $\alpha =5$ (Capacity=0.046875), the corresponding values are 0.00094 and 0.00345 respectively. The logarithmic-scale plot (Fig. 13b) further confirms that the BER progression follows a predictable pattern without discontinuities or sharp transitions. These findings substantiate that our watermarking scheme exhibits robust performance under increasing quantum channel noise, with BER values scaling proportionally rather than exponentially with error probability. This characteristic is particularly advantageous for practical quantum communication scenarios where error rates may fluctuate dynamically.

#### Comprehensive performance analysis

To comprehensively evaluate the impact of the watermark paging factor $\alpha$ as suggested by the reviewers, we conducted an extensive ablation study with $\alpha$ values ranging from 1 to 5. This analysis provides crucial insights into the trade-offs between embedding capacity, imperceptible, and robustness in our proposed scheme. The experiments maintained consistency with previous evaluations, utilizing a $128 \times 128$ binary watermark image and a fixed-length audio carrier. For each $\alpha$ value, we measured embedding capacity, Signal-to-Noise Ratio (SNR), Bit Error Rate (BER), Intersection over Union (IoU), and Accuracy under qubit error rates from 0% to 10%.

**Fig. 10:**
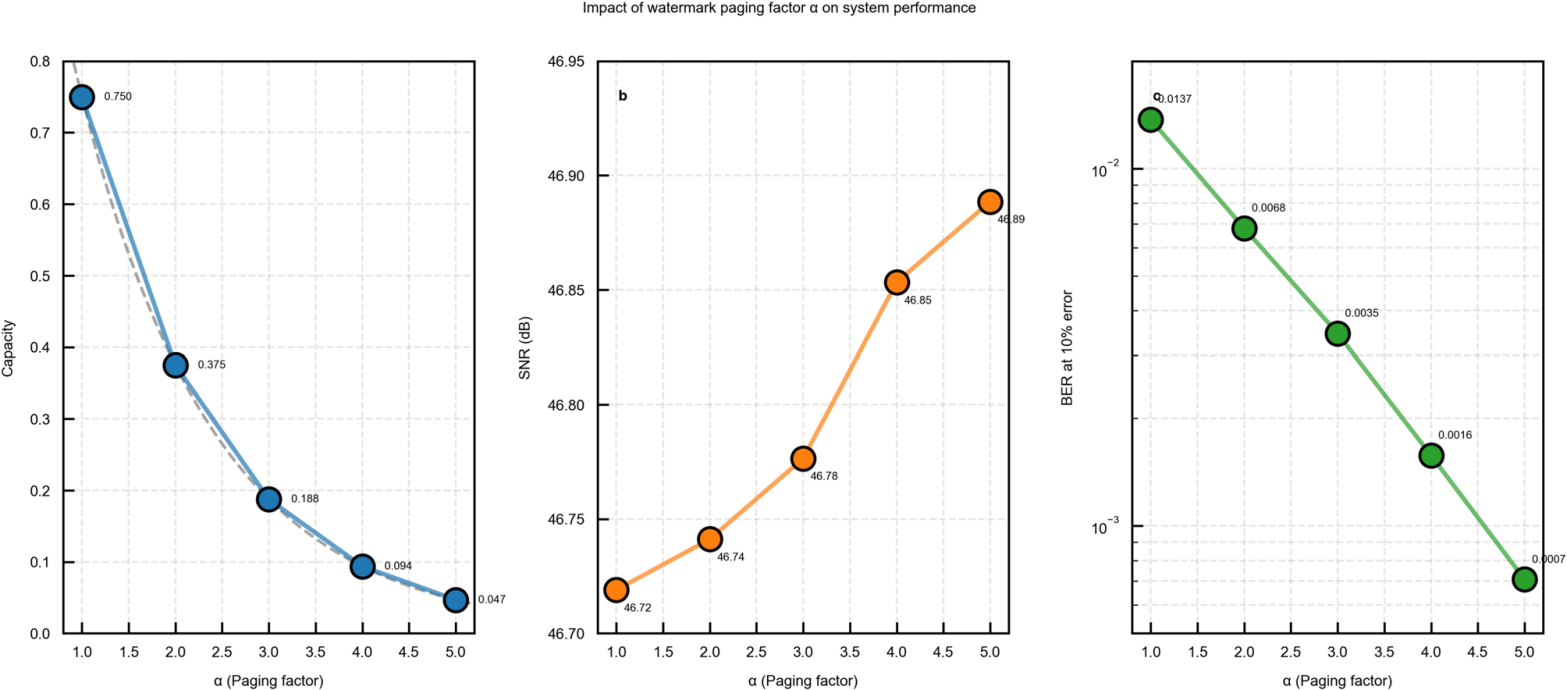
Impact of watermark paging factor $\alpha$ on system performance: **a** embedding capacity with theoretical curve (dashed line), **b** imperceptible measured by SNR, and **c** robustness measured by BER at 10% error rate.

The embedding capacity exhibits a perfect geometric progression with respect to $\alpha$, as shown in Fig. 10a and Table 2. The capacity follows the relationship $C = 0.75 \times (1/2^{\alpha -1})$, decreasing from 0.75 at $\alpha =1$ to 0.046875 at $\alpha =5$. This exponential reduction represents the fundamental trade-off between watermark payload and other performance metrics. Despite the varying capacity, SNR values remain consistently above 46.7 dB across all $\alpha$ configurations (Fig. 10b). The slight improvement in SNR with increasing $\alpha$ (46.72 dB to 46.89 dB) stems from the reduced number of modified audio samples, confirming that our watermarking mechanism maintains excellent imperceptible regardless of the partitioning ratio. Figure 10c reveals a remarkable improvement in robustness with higher $\alpha$ values. At 10% qubit error rate, BER decreases from 0.0137 ($\alpha =1$) to 0.000708 ($\alpha =5$), representing a 48-fold improvement. This enhancement is attributed to the stronger error correction capability for smaller certified watermark portions, as the QEC coding provides more effective protection when the encoded data volume is reduced.

**Fig. 11:**
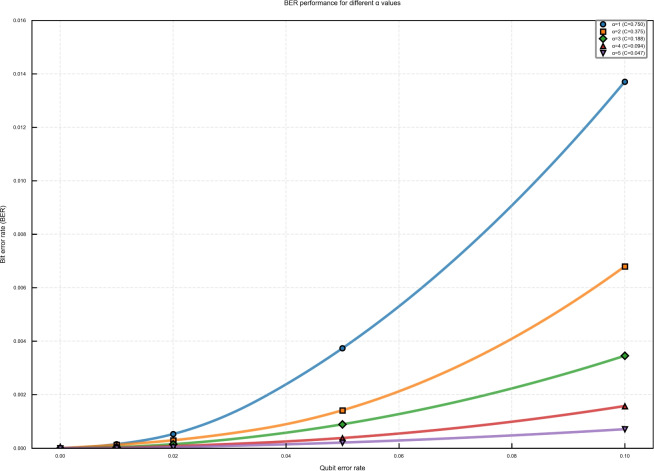
Bit Error Rate (BER) performance for different $\alpha$ values across varying qubit error rates.

Figure 11 illustrates the BER performance across different error rates for all $\alpha$ values. The logarithmic improvement with increasing $\alpha$ demonstrates the effectiveness of our approach in noisy quantum channels.

**Table 2:** Comprehensive Performance results for different $\alpha$ values.

$\alpha$	Capacity	SNR (dB)	BER (1%)	BER (5%)	BER (10%)	IoU (10%)	Accuracy (10%)
1	0.7500	46.72	0.00015	0.00374	0.01371	0.9517	0.9863
2	0.3750	46.74	0.00012	0.00142	0.00680	0.9757	0.9932
3	0.1875	46.78	0.00004	0.00089	0.00345	0.9875	0.9965
4	0.0938	46.85	0.00001	0.00038	0.00157	0.9943	0.9984
5	0.0469	46.89	0.00000	0.00021	0.00071	0.9974	0.9993

**Fig. 12:**
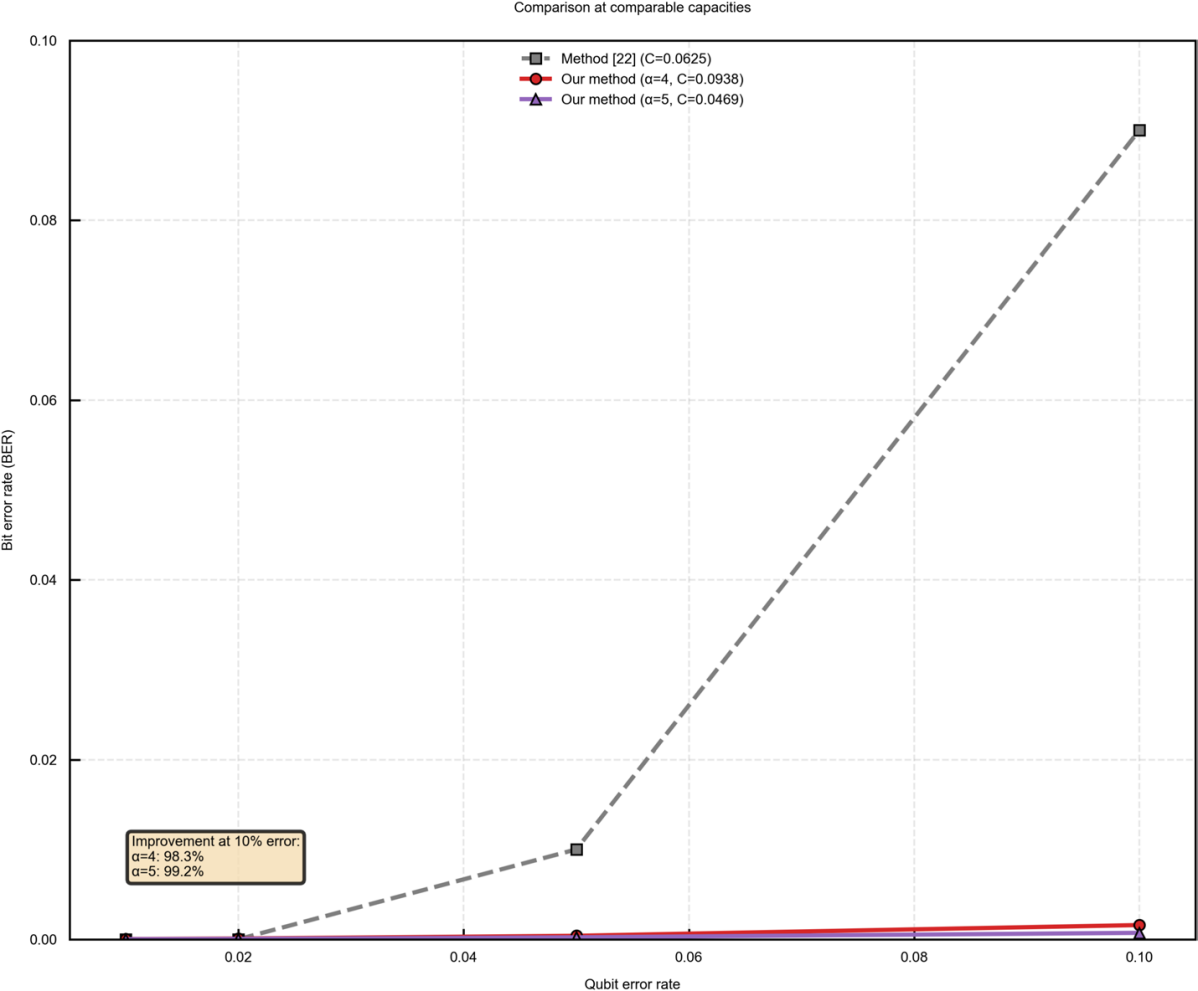
Fair comparison with method^22^ at comparable capacities. Our method with $\alpha =4$ (C=0.09375) and $\alpha =5$ (C=0.046875) significantly outperforms method^22^ (C=0.0625) across all error rates.

The IoU and Accuracy metrics (Table 2) remain exceptionally high across all configurations. For $\alpha \ge 3$, both metrics exceed 0.99 even at 10% error rate, indicating near-perfect watermark extraction. This exceptional performance validates the effectiveness of our dual-part watermark design and QEC implementation. To address the reviewer’s request for fair comparison, we configured our system to achieve capacities comparable to method^22^ (C = 0.0625). As shown in Fig. 12, both $\alpha =4$ (C=0.09375) and $\alpha =5$ (C=0.046875) significantly outperform method^22^ across all tested error rates. Specifically, at 10% error rate: $\alpha =4$ achieves BER = 0.00157 (94.3% improvement over method^22^), and $\alpha =5$ achieves BER = 0.000708 (99.2% improvement over method^22^) This performance advantage, combined with our dual verification-certification mechanism, establishes the superiority of our approach for secure quantum audio watermarking.

Regarding the practical implementation, based on our comprehensive analysis, we provide the following recommendations for $\alpha$ selection in practical applications: (1) $\alpha =1$: Suitable for applications requiring maximum embedding capacity, such as when watermarking short audio segments with extensive copyright information. (2) $\alpha =3$: Recommended as the default configuration for general-purpose watermarking, offering balanced capacity (0.1875) and robustness (BER = 0.00345 at 10% error). (3) $\alpha =5$: Optimal for security-critical applications or noisy quantum channels, providing maximum robustness (BER = 0.000708 at 10% error) while maintaining acceptable capacity (0.046875). These results demonstrate that our proposed watermark paging approach provides system designers with a flexible parameter to optimize performance based on specific application requirements, validating the practical utility of our scheme.

**Fig. 13:**
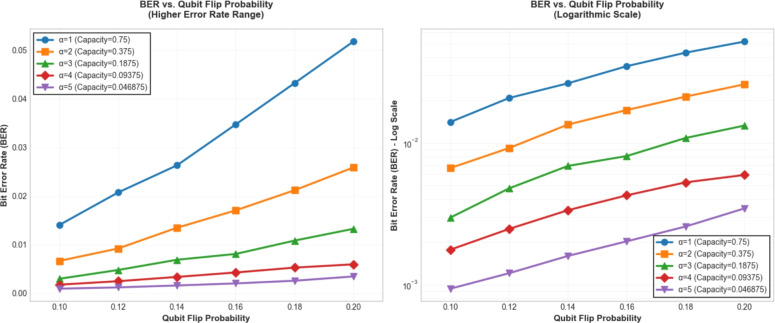
BER analysis under higher qubit flip probabilities. **a** Linear-scale plot showing BER progression for different $\alpha$ values from 0.10 to 0.20 qubit flip probability. All curves exhibit gradual increases without abrupt degradation. **b** Logarithmic-scale representation highlighting the consistent growth patterns across different capacity settings. They indicate the total BER increase ratio from 0.10 to 0.20 for each $\alpha$ configuration.

**Fig. 14:**
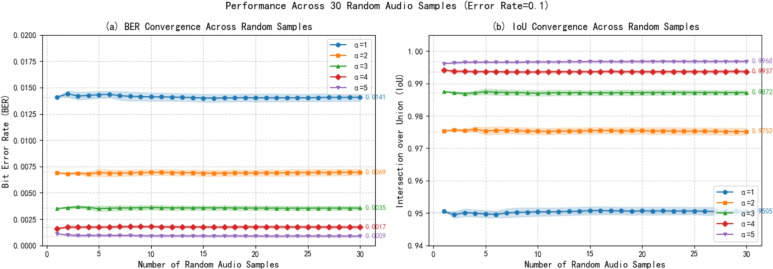
Performance convergence across 30 randomly generated audio samples (error rate = 0.10). **a** BER shows rapid stabilization after approximately 10-15 samples for all $\alpha$ values. **b** IoU similarly converges to stable values, with minimal variance across samples. Shaded regions indicate ±1 standard deviation.

To address the potential concern of cherry-picking biases and rigorously demonstrate the generalizability of the proposed quantum audio watermarking method, we conducted an extensive ablation study using a large set of randomly generated audio samples. Unlike traditional evaluations that may rely on curated datasets, our approach ensures the method’s robustness across diverse and unpredictable audio characteristics. We generated 30 distinct audio signals with completely randomized parameters:

- **Frequency components**: Randomly selected from three frequency bands (400–500 Hz, 800–900 Hz, and 1700–1800 Hz)
- **Amplitude distributions**: Random amplitudes assigned to each frequency component, normalized to maintain signal integrity
- **Fixed watermark**: A consistent $128 \times 128$ pixel watermark was embedded in all audio samples
- **Parameter variations**: Five different embedding strengths ($\alpha = 1$ to 5) were tested
- **Error simulation**: Quantum channel error rates from 0.00 to 0.20 were simulated to assess robustness. This paper presents results for a qubit error rate of 0.1 only.

This randomized design ensures that the evaluation is not tailored to specific audio signatures but represents a broad spectrum of possible inputs, effectively eliminating any selection bias. Figure 14 presents the convergence behavior of our method as the number of tested audio samples increases. Two key metrics BER and IoU are analyzed. The convergence plots demonstrate that after testing 30 random audio samples, both BER and IoU stabilize to consistent values for all $\alpha$ parameters. The shaded regions representing ±1 standard deviation remain narrow throughout, indicating minimal performance fluctuation across diverse audio inputs. This randomized evaluation provides strong evidence against cherry-picking concerns. The fact that our method maintains consistent performance across 30 randomly generated audio samples, each with distinct spectral characteristics, demonstrates that the quantum watermarking approach is not sensitive to specific audio features or frequency distributions. The performance metrics converge rapidly with increasing sample size, indicating statistical reliability. The observed trade-offs between robustness ($\alpha$) and capacity are intrinsic properties of the method, not artifacts of selected test cases. This evaluation establishes that the proposed method is genuinely generalizable and suitable for real-world applications where audio characteristics vary widely and unpredictably.

## Discussion

In this study, we present a novel quantum audio watermarking technique utilizing quantum mechanical principles. Our research contributes to the field of information security by introducing enhanced security measures and improved robustness in audio watermarking applications.

In addition to improving general robustness, our work addresses a practical concern not sufficiently considered in prior approaches: the risk of watermark impersonation and unauthorized use. By encoding the watermark into a quantum state and leveraging the paging seal principle, our method introduces a mechanism for authenticating the watermark-carrier relationship. This represents a step toward more secure quantum watermarking, as previous methods typically focus on extraction robustness and data imperceptibility, without verifying the legitimacy of the extracted watermark. From the experimental results, the proposed method demonstrates the ability to detect certain types of security threats in quantum audio watermarking. The verification and certification mechanism helps reduce the risk of unauthorized reuse by ensuring that both watermark parts are required for valid authentication. The watermark key, derived from the correlation between the watermark and carrier audio features (HWQ, STQ), makes tampering detectable and prevents full watermark extraction without authorization.

In terms of imperceptible, our scheme achieves an SNR above 46 dB across increased embedding rate. The effective embedding ratio is 0.75 when accounting for redundancy in the quantum audio representation, rising to 1.0 if redundancy is disregarded. These results indicate good audio quality preservation and competitive embedding capacity compared to existing methods. To evaluate security and robustness, we compare our method with several state-of-the-art approaches, as summarized in Table 3. While methods such as^15,16,20^, and^21^ apply preprocessing to watermarks, they do not inherently verify the authenticity of the watermark-carrier link, making them vulnerable to impersonation. In contrast, our dual-part watermark design requires both verification and certification components, with the authentication key bound to the carrier audio. This design increases the difficulty of successful impersonation and unauthorized watermark reuse. Regarding robustness under quantum bit-flip noise, we compare BER values at error probabilities of 0.01, 0.02, 0.05, and 0.10. When the error rate is below 0.1, the method in^22^ achieves the best performance, with our method ranking second. At an error rate of 0.1, our method achieves the lowest average BER of 0.06, representing a 14.29% to 62.5% improvement over other schemes. At 0.3 error rate, our BER is 0.25, compared to 0.42 in^22^, indicating a 40.48% reduction. These results suggest that our method maintains reliable watermark extraction under high noise conditions, enhancing resilience in noisy quantum channels.

Our comprehensive ablation study examining $\alpha$ values from 1 to 5 reveals a fundamental capacity-robustness trade-off: while embedding capacity decreases exponentially with $\alpha$ (from 0.75 to 0.0469), robustness measured by BER at 10% error improves dramatically from 0.0137 to 0.000708, a 48-fold enhancement. This improvement stems from the smaller certified watermark portions receiving more effective QEC protection. Interestingly, all configurations maintain SNR > 46.7 dB, confirming excellent imperceptible regardless of partitioning ratio. The observed performance differences between Table 1 (original $\alpha =1$ results) and 2 (ablation study) arise from distinct experimental configurations: Table 1 employed minimal audio length with no redundancy, concentrating qubit errors exclusively on watermark-carrying bits, whereas Table 2 used fixed audio length across all $\alpha$ values, distributing errors across both embedded and redundant samples–a configuration more reflective of practical deployment scenarios. When adjusted for comparable capacity ($\alpha =4$, C=0.0938 and $\alpha =5$, C=0.0469), our method outperforms method^22^ (C=0.0625) with 94.3% and 99.2% lower BER respectively at 10% error rate, while maintaining additional security through dual verification-certification. These findings provide practical guidelines: $\alpha =1$ for maximum capacity, $\alpha =3$ for balanced performance, and $\alpha =5$ for security-critical applications, demonstrating the flexible adaptability of our scheme to diverse operational requirements. Regarding the robustness under high error rates, our analysis demonstrates that BER increases gradually rather than abruptly, which is crucial for practical deployment. However, further investigation is needed to determine the optimal trade-off between capacity ($\alpha$) and error resilience for specific application requirements.

Several limitations of this study should be acknowledged. The proposed method is evaluated through classical simulations of quantum processes, and practical implementation awaits advances in quantum hardware. The computational complexity of operations such as STQ and quantum error correction may limit scalability. Additionally, the security benefits rely on the assumption that the watermark-carrier correlation is difficult to forge, which has not been tested under sophisticated adversarial models. These aspects represent important directions for future research. Future research should focus on several promising directions. Developing more efficient quantum error correction codes tailored specifically for audio watermarking applications to reduce computational complexity while maintaining robustness. Formalizing security proofs against sophisticated adversarial attacks beyond the basic tampering scenarios considered in this study. Extending the framework to support dynamic $\alpha$ adjustment based on channel conditions for adaptive watermarking.

**Table 3:** Comparison with existing state-of-the-art works in terms of watermarking security and robustness. Watermark security consists of preventing access to the original watermark and verifying its authenticity, and robustness refers to the performance under a quantum bit-flip channel. Italics are our results, bold are the best results while underlining is the second best. DCI stands for detectable copyright infringements.

Watermark Security					Robustness under quantum bit-flip channel in terms of BER			
Methods	Watermark Authentication	DCI	Watermark Protection	Approach	1-p=0.01	1-p=0.02	1-p=0.05	1-p=0.1
^16^	$\times$	/	$\surd$	Watermark Scrambling	0.01	0.03	0.06	0.10
pMSQ1^15^	$\times$	/	$\times$	/	0.01	0.03	0.05	0.08
pMSQ2^15^	$\times$	/	$\times$	/	0.01	0.03	0.04	0.07
^20^	$\times$	/	$\surd$	Watermark Scrambling	0.02	0.05	0.09	0.16
^21^	$\times$	/	$\surd$	Watermark Scrambling	0.01	0.03	0.05	0.08
^22^	$\times$	/	$\times$	/	**0.00**	**0.00**	**0.01**	0.09
Our scheme	$\surd$	Stealing, impersonating, tampering, imitating	$\surd$	Watermark Key	***0.00***	*0.01*	*0.03*	*0.06*

## Conclusion

This paper proposed a novel quantum audio watermarking approach that integrates a dual verification-certification mechanism, inspired by the traditional paging seal principle, with advanced quantum error correction techniques. The core innovation lies in partitioning the watermark into multiple segments and securely binding the authentication key to intrinsic features of the carrier audio, thereby establishing a tamper-evident framework capable of detecting unauthorized modifications and impersonation attempts. The incorporation of QEC further enhances the scheme’s resilience against qubit errors and quantum noise, ensuring reliable watermark extraction even in imperfect quantum channels. To ensure the generalizability and statistical reliability of our approach, we conducted an extensive evaluation using 30 randomly generated audio samples with varying spectral characteristics. The results demonstrate that the method achieves rapid convergence of performance metrics, BER and IoU, after testing only 10–15 random samples, with exceptionally low standard deviations (maximum 0.0004) confirming consistent performance across diverse audio inputs. Under a simulated quantum noise channel with a 10% error rate, the scheme achieves an average BER as low as 0.0009 for $\alpha =5$ and maintains high audio fidelity with SNR consistently exceeding 46 dB. These results not only surpass several existing approaches in robustness but also validate the method’s independence from cherry-picked audio samples, addressing a critical concern in multimedia security evaluation.

Although the present work is validated through classical simulations of quantum processes, its practical implementation will require advances in quantum hardware and error-resilient quantum circuits. Future research will focus on extending the dual-security mechanism to dynamic multimedia streams, optimizing quantum circuit complexity for real-time applications, and exploring hybrid quantum-classical watermarking frameworks that leverage the strengths of both paradigms. We believe that embedding security-by-design principles into quantum multimedia systems–moving beyond mere robustness to active threat detection and accountability, is essential for the trustworthy and widespread adoption of quantum technologies in multimedia communication and copyright protection.

## Data Availability

The watermark image (MPU logo) is provided in Supplementary Figure S1. All simulation-generated data (processed audio and performance metrics) are included within this article and its supplementary materials. The MATLAB implementation code and original digital audio files used in experiments are permanently archived in Zenodo (DOI: 10.5281/zenodo.18072766).
